# Annotations

**Published:** 1889-05-04

**Authors:** 


### Annotations.

**A Street Ambulance.**

Hoaxing the press is a favourite amusement of some people, but it is rarely successful nowadays, owing to the excellence of the editorial arrangements. The *City Press* has, however, been hoaxed, and has succeeded in hoaxing the *Chronicle*, which reproduced its extraordinary paragraph on the above scheme on 29th ult. It is there stated that "Sir Sidney Waterlow has initiated a movement for establishing in all parts of London ambulances for the conveyance to the hospitals of all people who may meet with street accidents," etc. Anything further from the facts could scarcely be imagined, as the columns of the *Chronicle* show. The street ambulance proposals were initiated by The Hospitals Association, and the scheme is the outcome of the labours of Mr. Ryan, secretary to St. Mary's Hospital. When it had been adopted, and the whole of the funds necessary for its establishment had been obtained, a committee was appointed by The Hospitals Association, of which Sir Sidney Waterlow became a member. Sir Sidney was subsequently appointed chairman of this committee, and since that time has taken a great interest in the work, and has recently rendered material assistance in perfecting the organisation. We are sure Sir Sidney Waterlow would be the last person to claim credit for work with which he had nothing to do, and, bearing in mind recent experiences with reference to similar claims, we think it desirable to place these facts on record. Philanthropists, of all people, ought to take care to give honour to whom honour is due, and to no one else.

**Religious Intoxication.**

There is no doubt that human beings often become intoxicated, although they may be total abstainers. Indeed, asceticism in one direction often tends to excess in another. Whilst we are grateful for the improvements which have taken place of late in the methods of observing Good Friday and Easter Sunday, we cannot conceal from ourselves certain dangers attaching to modern religious observances. Some mothers very rightly object to their daughters, for example, spending the whole of Good Friday in church without proper food or sustenance of any kind. One lady states that her daughter commenced at six o'clock in the morning, and remained in church until five o'clock in the afternoon. After an hour's interval, she returned to the church again, and remained there until late at night. Recently, a service, entitled "Tenebræ," has been started by certain of the clergy, which consists of the gradual extinguishment of all lights as the service proceeds, until the whole building is enveloped in darkness. Mothers, quite rightly, object to the youth of both sexes attending such services, and they regard them as calculated to reintroduce all the vice and evil attaching to revivalist services of past times. What with communions from five a.m. onwards, watchings, confessions, church duties, and the multifarious services which certain clergymen now endeavour to induce young ladies to undertake, the happiness of many families and individuals is becoming undermined. For a clergyman who is bound by solemn vows to inculcate morality, sobriety, moderation, virtue, and a tender regard for others, to use his church for organising and enforcing religious excess in every direction is to prove himself an unfaithful steward, dangerous to the best interests of young and old alike. If some check is not put to the unwholesome excitements fostered by certain of the clergy, many a daughter's health will be undermined, if her life is not ruined, through the influence of her so-called father-in-God. In the interests of morality, of sobriety, of health, of sober sense, and of the national life, we protest against religious excess of all kinds, and we look upon the clerical intoxication here referred to as a social danger, which, if unchecked, must soon result in the degradation of many of the rising generation.

**Shock.**

Few of us realise the meaning of the word "shock." In medicine, it conveys to the physician grave apprehensions and serious misgivings for the life of his patient. In cases of serious injury arising from an accident, or the assault of wild beasts, shock acts as an anæsthetic after the event, and sometimes during its course. Thus, travellers, from Livingstone downwards, have related that when struck by a tiger or lion they experienced sensations of peaceful contentment, and were unconscious of pain when the beast proceeded to maul, and even to consume, an arm or some other part of the body. To the healthy and vigorous, the meaning of the word "shock" may come home forcibly in the course of a visit to the dentist. The healthy man, strong and robust, who goes to his dentist to have a tooth stopped, and is told unexpectedly that a four-fanged molar must be taken out at once, immediately realises the meaning of shock. He is surprised to find that the announcement creates a sickly feeling. Although he may possess ordinary courage, the sudden insistence of the infliction upon him of an unknown amount of pain causes a failure of courage for the moment, followed by the sickly feeling referred to, which his manhood resents. His experiences are, however, but the result of a kindly provision of nature, which, by depressing the nervous system, secures a lessened sensibility to pain. It is well known that those who have to suffer pain continuously become almost habituated to it, and, after a time, it is only when they are seized by a sudden paroxysm that they experience much inconvenience from their continuous suffering. So shock, which to the strong and healthy means momentary loss of control, becomes in the seriously injured a narcotic force of such power that many regard it as the chloroform which nature has mercifully extended to the animal creation.

**Overfeeding.**

Excessive eating is not the most striking or the most widely prevalent fault of the present generation. On the contrary, moderation and even sometimes undue limitation in diet is the prevailing fashion. There are still, however, a considerable number of persons who habitually overeat at meals; and to such a few physiological hints may not be without their value. Dr. Reudon has been at the pains to make some careful investigations on the subject, and his results have recently been published. According to this observer, a not uncommon consequence of over-feeding is the development of a series of symptoms in many respects similar to those of typhoid fever. The temperature rises, there is a feeling of serious illness, the sleep is disturbed, the brain is incapacitated, and in severe cases the disability is complete. The cause of these symptoms is insufficient elimination, and an alteration in the blood brought about by the impregnation of the organism with accumulated waste products. In addition to these typhoid symptoms thromboses occur in the vessels, and what is known as spontaneous gangrene, or mortification of parts without any obvious or sufficient cause. Now, these are conditions of very marked danger, particularly the thrombosis and the spontaneous gangrene. There is danger to life here. The obvious remedy for such a series of evils is, of course, rest for the overworked digesting and eliminating organs. Both the quantity and the quality of the food must be so changed as to admit of the performance of easy digestion, perfect assimilation, and adequate elimination of waste. Lemonades and lemon juice are said to be of great service in diminishing the extreme craving for food; and this, from a limited experience, we can, to some extent, confirm. Milk, also, in moderate quantities is useful, and in certain cases skim milk would probably be best. It is not always found that the resting of the organs is sufficient. The fever may persist for a long time, and with it the feeling of very decided illness. Drugs of different kinds are then urgently demanded, and a competent physician should be consulted without delay.

